# Coherence, transformation, and breakdown in living systems: part II — predictions and implications for systemic health

**DOI:** 10.3389/fsysb.2026.1910994

**Published:** 2026-10-14

**Authors:** Andreas Nicolaides

**Affiliations:** Independent Researcher, Harrogate, United Kingdom

**Keywords:** biological coherence, CHORA hypothesis, systemic disease, entrenchment, evolutionary coherence, higher-order regulatory architecture, systemic health, transgenerational inheritance

## Abstract

Part I of this two-part series advanced the CHORA Hypothesis as a candidate account of biological coherence: how organised biological function is sustained, transformed, and lost within the organism and across the lineage over deep time. The hypothesis holds that a Common Higher-Order Regulatory Architecture (CHORA) operates as an integrated, hierarchically structured level of organisation across cells, tissues, organisms, and generations, sustaining within-organism coherence and evolutionary coherence alike. It holds further that the germline is the principal conduit through which the architecture is propagated, that the same architectural features which sustain biological coherence underlie the characteristic modes of its failure, and that disruption of the architecture at the organism level underlies the convergent signatures of systemic disease. This paper develops the empirical predictions and the systemic health implications that follow. Section 1 sets out six predictions under the commitments from which they follow. The first three follow from the architecture’s integrated operation: catastrophic rather than graded failure at architecturally central components; the concentration of lineage transitions at mid-hierarchical regulatory levels; and the bidirectional intergenerational consequences of perturbation at germline-soma coupled components. The fourth derives from germline priority in propagation and concerns intergenerational erosion under partial germline compromise, with somatic analogues at architecturally shared components. The fifth derives from the coherence-breakdown relationship and concerns the entrenchment of pathological regimes through feedback architectures repurposed under displaced upstream conditions. The sixth derives from disruption of the architecture at the organism level and concerns the convergence of systemic pathologies on shared signatures of architectural breakdown. Each prediction is examined against alternative accounts and the postdictive and prospective evidence bearing on it. Several predictions organise existing evidence under a single architectural account; others are mainly prospective. Section 2 draws out the implications for systemic health. Health and entrenched systemic disease are not opposing states but alternative regimes of the same biological-coherence-sustaining architecture. On this account, developmental origins, ageing, and intergenerational transmission are intelligible as expressions of a shared architectural logic. The deeper ontological question of what kind of thing this architecture is, together with the framework’s broader conceptual implications, is addressed in a forthcoming publication.

## Introduction

The question of how living systems sustain organised function not only across the lifespan of individual organisms but across the lifespan of lineages was developed in Part I of this two-part series. There this general property was termed biological coherence, and its lineage-scale expression, the principal concern of Part I, was termed evolutionary coherence. Part I argued that the patterns visible when biological coherence is examined directly are not fully accounted for by the standard frameworks of evolutionary biology and biomedicine. It developed the suggestion that these patterns are intelligible as the evolutionary behaviour of a common higher-order regulatory architecture sustaining biological coherence over deep time in eukaryotic and especially metazoan biology, and named this framework the CHORA Hypothesis. CHORA names not a further regulatory network but the organisational level at which the five networks Part I surveys jointly operate as one architecture spanning both organism and lineage.

The hypothesis carries four commitments. The first is that this higher-order regulatory architecture operates as an integrated level of organisation across cells, tissues, organisms, and generations. This integration is itself hierarchically structured: within each architecture, some components occupy an architecturally central position, whose disruption threatens the coherence of the system as a whole, while others occupy more peripheral positions, whose modification is comparatively well tolerated. The molecular substrates examined in Part I are understood as expressions of a common organisational character rather than as architecturally independent phenomena. The second is that the germline occupies a specifically privileged position in the propagation of the architecture, as the principal conduit through which the conditions for evolutionary coherence are re-established in each new generation. The third is that the same architectural features that sustain biological coherence also underlie the characteristic modes of its failure, with robustness, ordered transformation, and the conditions of breakdown understood as consequences of the same architectural logic operating under different conditions. The fourth is that disruption of the architecture at the organism level underlies the convergent signatures of systemic disease, with health and entrenched systemic disease understood as alternative regimes of the same biological-coherence-sustaining architecture rather than as opposing biological states.

The argument of Part I proceeded by abductive inference: it identified the requirements an account of evolutionary coherence must meet, surveyed molecular evidence demonstrating that architectures meeting those requirements are concretely present in living systems, and developed a systems model characterising how such architectures operate, advancing the CHORA Hypothesis on that basis.

The convergence of chronic conditions with differing proximate causes is worth restating briefly, since it shapes the predictions developed below. The gap lies between the patterns biological coherence displays and what the standard frameworks predict. Evolutionary biology’s standard account explains change through variation, inheritance, and selection (Futuyma and Kirkpatrick, 2022). The lineage-scale patterns central to this series are the deep conservation of regulatory architecture despite divergent selective regimes, catastrophic rather than graded failure at particular components, and transitions concentrated at particular hierarchical levels. The standard account has engaged these through auxiliary hypotheses as each became apparent: punctuated equilibria addressed the tempo of morphological change (Eldredge and Gould, 1972); canalisation and developmental constraint addressed the structured availability of variation (Wagner, 2005); and the Extended Evolutionary Synthesis addressed the role of developmental process in shaping evolutionary outcome (Laland et al., 2015). Contemporary biomedicine has engaged a structurally similar gap at the organism scale. The convergence of chronic conditions with differing proximate causes (cancer, autoimmune disease, neurodegeneration, age-related decline) on shared signatures of regulatory dysfunction is not predicted by a component-level account of disease. It has been engaged through auxiliary frameworks including network medicine (Menche et al., 2015) and the hallmarks-of-cancer and hallmarks-of-ageing literature (Hanahan and Weinberg, 2011; López-Otín et al., 2013). Each auxiliary supplements the framework employing it without displacing its central commitments, and points toward, without fully articulating, the architectural level Part I developed as the CHORA Hypothesis.

Two existing bodies of work bear on this gap, in different ways. Evolutionary medicine (Nesse and Williams, 1997; Nesse et al., 2010) has long argued that evolutionary biology is underused in medicine, but its links are adaptationist, concerned with why selection leaves the body vulnerable, not with the architectural level proposed here. Systems biology comes closer: it establishes that biological robustness links evolvability and disease (Kitano, 2004), though as a general property, not a specific conserved architecture. The present account proposes something more specific than either: that the regulatory architecture whose conservation sustains coherence across the lineage is the same architecture that fails, in characteristic regimes, to produce the convergent signatures of systemic disease. To our knowledge, no existing framework connects evolutionary coherence and systemic disease at this architectural level. The six predictions below are read against this gap (examined in full in Part I, Section 3). Where possible, these tensions are traced to a dispute within the relevant field.

This paper develops the consequences of the hypothesis in two domains. The first is empirical: Section 1 sets out six predictions following from the four commitments, examined against what alternative accounts predict and the evidence bearing on them. The predictions are offered in two registers. Some are postdictive: the hypothesis renders coherent, under a single architectural account, observations already established but not previously articulated as expressions of a shared organisational logic. Others are prospective: the hypothesis predicts findings not yet established that future investigation could test. The evidential standing of each prediction, and the case for postdictive unification specifically, is assessed in the Discussion.

The second domain concerns systemic health. This is built directly into the framework’s fourth commitment (that disruption of the architecture at the organism level produces the characteristic forms of systemic disease), not derived as a downstream application of an evolutionary framework whose primary domain lies elsewhere. Section 2 develops the implications that follow. It does not attempt a medical account of any particular condition, nor does it replace the mechanistic work required to understand specific pathologies. Its aim is more limited: to indicate what becomes conceptually visible when systemic health is understood through the architectural framework developed in Part I and refined through the predictions of Section 1.

The empirical and health-relevant case must stand or fall on its own merits, and that is what this paper examines. Several of the predictions developed below concern molecular systems (the piRNA pathway, centromeric architecture, the dynamics of repetitive DNA) whose rapid evolution is often read in selectionist terms, as Red Queen dynamics driving an arms race between host regulation and genomic conflict. The architectural account does not dispute that such selection might occur; it disputes that the arms race explains the cross-domain recurrence of the architectural signature at issue. This relation between the architecture and natural selection is taken up in a forthcoming companion paper on the framework’s philosophical foundations, a separate work standing outside the present two-part series. The concern of the present series, throughout, is the empirical case for the hypothesis and its implications for systemic health. The predictions below are framed so that their discriminating force does not depend on it: each is assessed against what the architectural and selectionist accounts respectively predict, and stands on that comparison.

## The six predictions

Six predictions are developed below, grouped by the commitment from which each follows. The first three follow from the architecture’s integrated operation: catastrophic rather than graded failure at architecturally central components (Prediction 1), the concentration of lineage transitions at mid-hierarchical levels (Prediction 2), and bidirectional intergenerational consequences at germline-soma coupled components (Prediction 3). The remaining three follow respectively from germline priority in propagation, the coherence-breakdown relationship, and disruption at the organism level: intergenerational erosion under partial germline compromise with somatic analogues (Prediction 4); the entrenchment of pathological regimes through repurposed feedback architectures (Prediction 5); and the convergence of distinct systemic pathologies on shared architectural signatures (Prediction 6). Each prediction is assessed against the postdictive and prospective evidence bearing on it, the predictions of alternative accounts, and the verdict criteria for further testing. Figure 1 illustrates this structure, mapping the six predictions onto the four commitments from which they follow.

**FIGURE 1:**
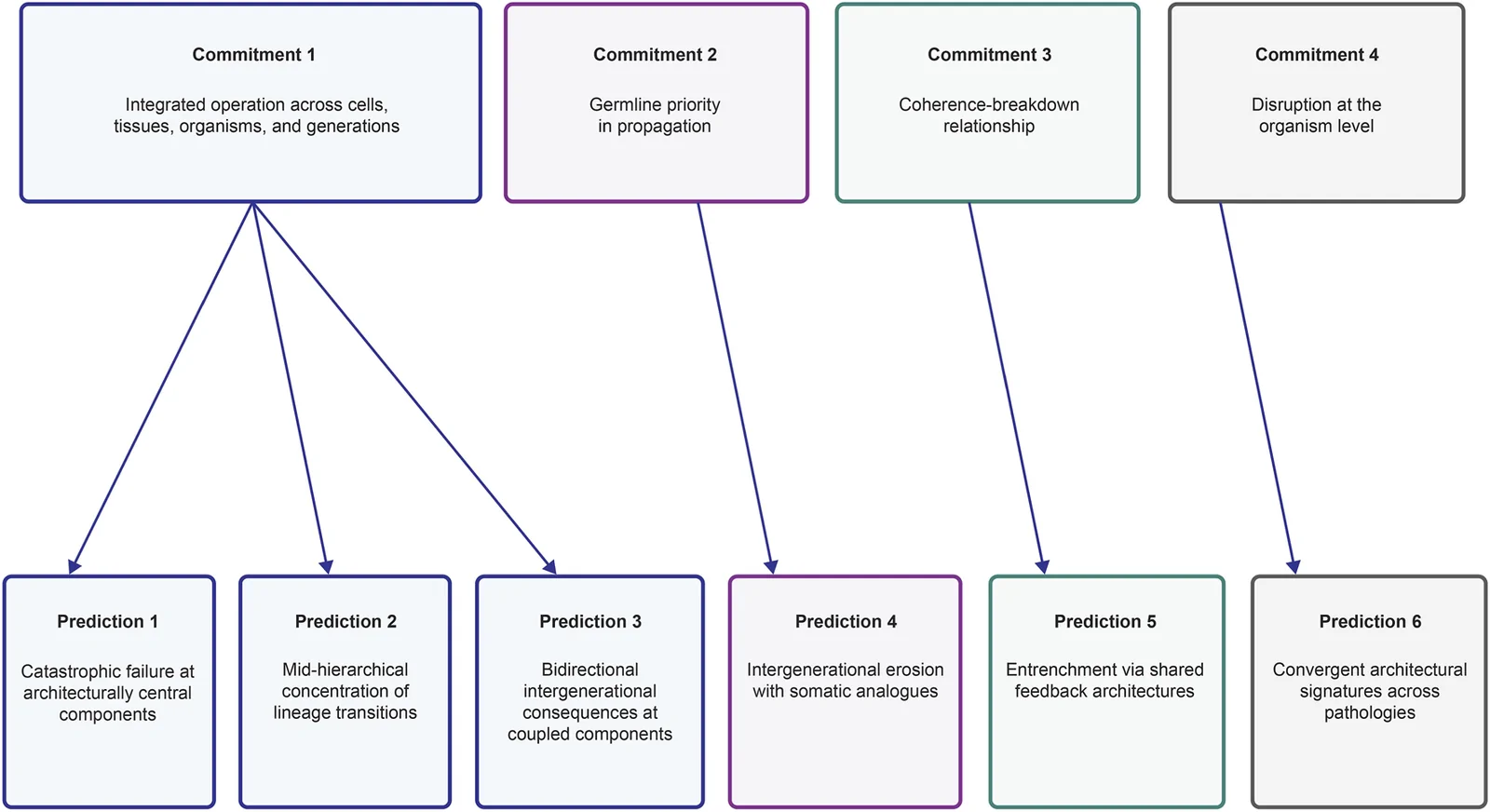
The six predictions mapped onto the four commitments of the CHORA Hypothesis. Predictions 1, 2, and 3 follow from the architecture’s integrated operation across cells, tissues, organisms, and generations; Prediction 4 from germline priority in propagation; Prediction 5 from the coherence-breakdown relationship; and Prediction 6 from disruption of the architecture at the organism level. The grouping follows the structure of Section 1 rather than architectural level (kernel, mid-hierarchical, or peripheral position), since only Predictions 1 and 2 concern architectural centrality specifically; the remaining predictions follow from the other three commitments and are not organised by hierarchical position.

### Predictions following from the integrated operation of the architecture across cells, tissues, organisms, and generations

Three features follow from the hypothesis’s first commitment (see Introduction): the failure topology of components across the architecture (Prediction 1), the architectural location of lineage transitions (Prediction 2), and the consequences across the germline-soma axis of perturbations to coupled components (Prediction 3).

#### Prediction 1: Catastrophic rather than graded failure modes at architecturally central components

Perturbations to deeply embedded regulatory components (gene regulatory network (GRN) kernels, core germline regulatory systems, and centromere identity architecture) should exhibit predominantly catastrophic rather than graded failure modes. Intermediate states between functional and non-functional configurations should be absent, unstable, or non-viable. This is the prediction’s content, stated here in advance of the evidence bearing on it; the postdictive support for it, and the general mechanism (bistable, positive-feedback network dynamics) that would produce this pattern, are set out below.

##### Discriminating force

The prediction discriminates against alternative accounts on two counts: the topology of failure (the first three features below) and the pattern of conservation (the fourth). First, catastrophic failure should concentrate at architecturally central nodes (kernels, core germline regulatory systems, centromere architecture) — components occupying a structurally indispensable position within the regulatory hierarchy—while peripheral components continue to show the graded responses to perturbation characteristic of variation that selection can act on through small fitness differences. This asymmetry is not predicted by gradient-selection accounts, in which a component’s conservation tracks the strength of selection on its phenotypic output rather than its structural position. Second, this concentration should hold across phylogenetically vast distances and across regulatory systems with very different phenotypic outputs: kernels controlling cardiogenesis, gut specification, and body-axis patterning should each exhibit the same topology despite very different selective regimes. Third, it should appear at any further architecturally central sites the framework would identify, such as meiotic recombination or nucleolar chromatin systems, not only the three sites already characterised. Fourth, conservation of central components should track architectural centrality rather than the strength of selection on their outputs.

##### Postdictive evidence

This prediction is well supported by convergent evidence at each of the three architecturally central sites the framework identifies, though these cases are not typically presented together as a unified consequence of a single architectural logic. In the developmental GRN literature, kernels stabilising body-plan organisation are characterised by recursive positive-feedback wiring such that interference with any constituent gene results in developmental catastrophe rather than graded reduction in performance (Davidson and Erwin, 2006; Peter and Davidson, 2011): the endomesoderm specification network in sea urchin development, the heart-specification kernel conserved across bilaterians, and the dorsoventral patterning kernel in arthropods each exhibit qualitative loss of the specified outcome on perturbation, not a slightly compromised version of it. This threshold-dependent character is consistent with general results from the bistability literature, where networks built from positive-feedback motifs produce switch-like rather than graded responses to perturbation (Ferrell, 2002).

In the germline, disruption of core piRNA-pathway components does not yield a mildly compromised germline that still functions adequately for transmission; it produces transposable-element derepression, accumulation of DNA damage, and cascading germline collapse rather than graded decline (Brennecke et al., 2007; Malone and Hannon, 2009; Czech and Hannon, 2016). The Mortal Germline phenotype examined under Prediction 4 is the across-generation expression of the same architectural fragility: partial compromise is tolerated initially, but the trajectory is toward collapse rather than toward a stable diminished state. In centromere maintenance, loss of CENP-A or substantial disruption of the chromatin architecture that propagates centromere identity produces chromosome mis-segregation and aneuploidy, with no graded intermediate of partial centromere function (McKinley and Cheeseman, 2016; Barra and Fachinetti, 2018). Centromere identity is a binary property of the chromatin region rather than a quantitative one: a centromere is either competent to assemble a functional kinetochore or it is not, and the transition between these states is sharp.

##### Comparison with alternative accounts

The architectural account predicts the catastrophic-failure topology at central nodes as a structural consequence of how such components are embedded in the architecture; the standard framework has instead engaged the same phenomena through auxiliary hypotheses introduced to accommodate findings it did not predict. A standard gradient-selection account would expect at least some graded viability across variants of architecturally central components, since its selective trajectories (small, fitness-improving steps between functional variants) require viable intermediates. The persistent absence of such intermediates, across vast phylogenetic distances and outputs under very different selective regimes, is unsurprising on the architectural account but requires additional machinery on the gradient-selection account (a tension made explicit in Coyne’s (2006) published comment and Erwin and Davidson’s (2006) reply). A more sophisticated objection holds that this catastrophic-failure pattern is itself a by-product of selection for robustness, which can in principle make central components indispensable (Wagner, 2005). This comes closer, but the selection-for-robustness account does not predict the systematic concentration of catastrophic failure at central nodes specifically; it predicts only that some components will become canalised, remaining silent on the distribution of catastrophic versus graded failure across the genome. The architectural account makes the stronger structural prediction that the topology of catastrophic failure tracks architectural centrality, independently of lineage-specific selective pressures on outputs.

The prediction’s second discriminating claim concerns conservation rather than failure: conservation, too, tracks centrality. On a gradient-selection account, conservation of regulatory components broadly tracks the strength of selection on their phenotypic outputs: components whose outputs are under strong stabilising selection are conserved, components under relaxed selection drift. On the architectural account, conservation of architecturally central components is driven instead by their architectural centrality, because alteration is catastrophic regardless of the current selective environment, consistent with the pan-bilaterian conservation of the heart-specification GRN kernel across animals whose cardiovascular systems and ecological circumstances differ dramatically (Davidson, 2006; Peter and Davidson, 2011), and with the deep conservation of meiotic and reprogramming machinery across taxa with markedly different reproductive strategies (Hackett and Surani, 2013; Arter and Keeney, 2024).

##### Verdict criteria

Treat this as section level heading as per section 379 Findings extending the catastrophic-failure pattern to additional architecturally central components, in additional taxa, would support the prediction. The predicted asymmetry failing in either direction would weigh against the prediction: catastrophic failure distributed uniformly irrespective of centrality, or viable graded intermediates at architecturally central nodes, particularly if reproduced across multiple lineages. On the conservation claim specifically, systematic comparative analysis would discriminate: architecturally central components remaining conserved across lineages whose output-level selective pressures differ substantially would support the prediction, while their conservation varying with the strength of selection on outputs would weigh against it.

#### Prediction 2: lineage transitions concentrated at mid-hierarchical architectural levels

Major evolutionary reorganisations should coincide preferentially with alterations in mid-hierarchical regulatory components, rather than with modifications of kernel components (which are architecturally locked, as Prediction 1 sets out) or the most peripheral components (whose modification produces fine-grained phenotypic variation rather than reorganisation).

##### Discriminating force

Three features make the prediction discriminating against alternative accounts. First, lineage transitions, when localised at the regulatory level, should concentrate at mid-hierarchical components rather than being distributed uniformly across the hierarchy, a structural consequence of where the hierarchy admits modification at all. Second, this concentration should hold across clades whose ecological circumstances and selective pressures differ substantially: the same pattern should appear in arthropod body-plan diversification, vertebrate appendage evolution, and, beyond the metazoan focus of the present series, plant inflorescence diversification, despite the very different ecological contexts involved. Third, the trajectories accessible from a given regulatory configuration are structured by what mid-hierarchical modifications remain available once kernel-level constraints (the fact that kernel components cannot be modified without catastrophic failure) are taken into account.

##### Postdictive evidence

This prediction renders coherent, under a single architectural account, two patterns well-established but not typically presented together as expressions of a shared organisational logic. The first is the pattern of punctuation and stasis in the fossil record: stability of body-plan organisation across geological intervals, with relatively rapid transitions between configurations, was articulated by Simpson (1944) and developed as the central observation of punctuated equilibria (Eldredge and Gould, 1972; Gould, 2002). The pattern’s strength and generality remain debated, but extended stasis interrupted by structured transitions is broadly accepted as common across many clades (Hunt, 2007; Erwin, 2007). The second pattern is the architectural distribution of regulatory change associated with lineage transitions: Davidson and Erwin’s (2006) analysis of GRN evolution across animal body plans found that components most often modified during body-plan reorganisation are neither the architecturally locked kernels nor the most peripheral components, but the intermediate sub-circuits connecting them, termed plug-ins and input/output sub-circuits by Erwin and Davidson (2009). Independent work on other clades converges on the same picture: cis-regulatory modifications at intermediate-level transcription factors and signalling pathways account for a disproportionate share of regulatory change associated with morphological diversification (Carroll, 2008; Wittkopp and Kalay, 2012).

These two patterns are jointly explained by the structural account just sketched, applied here to tempo. Because kernel-level change is unavailable and peripheral change alone is inconsequential at the body-plan level, evolutionary change at that level should exhibit two regimes: extended intervals of accumulating peripheral variation that produce no visible reorganisation (observed as morphological stasis even where the lineage is far from genetically static), interrupted by relatively rapid reorganisations corresponding to mid-hierarchical regulatory change. The architectural account does not require punctuated equilibria’s strongest claim, that gradual change is the exception rather than the rule across evolutionary history generally: ongoing gradual variation at peripheral components is expected throughout, including during intervals of body-plan stasis, and is what the account attributes that stasis to. What the account does predict is the narrower claim that transitions in body-plan organisation specifically, arising from mid-hierarchical regulatory change, will exhibit this punctuated temporal signature; gradual, incremental change in body-plan organisation itself, rather than in the peripheral variation surrounding it, would weigh against the account.

##### Comparison with alternative accounts

The punctuated equilibria proposal is itself an auxiliary hypothesis of the standard evolutionary framework, in the sense developed in Part I, Section 3: it was introduced to accommodate a pattern the gradient-selection account did not predict. The exchange between Gould (1980) and Charlesworth et al. (1982) makes this tension explicit. Its substantive proposal (that stasis-and-transition reflects allopatric speciation rates and founder effects) does not predict the architectural location of the regulatory changes associated with transitions. The gradient-selection account, taken on its own, makes no specific prediction about that location either: its expectation is only that regulatory modifications correlate with where selection has been strong enough to drive them, which is compatible with the observed pattern but does not predict it. On the gradient-selection account, the mid-hierarchical concentration of transitions is a contingent outcome of where selection has acted; on the architectural account, it is a structural consequence of the hierarchy itself, holding regardless of lineage-specific selective history.

##### Verdict criteria

Systematic comparative analysis confirming the mid-hierarchical concentration of lineage transitions across additional clades and transition types would extend the postdictive support. Transitions found to concentrate at peripheral components, which produce fine-grained morphological variation rather than structural reorganisation, would weigh against the prediction, as would transitions found to concentrate at kernel components. Findings that the concentration varies with selective regime, appearing in some clades but not others, would weigh against the prediction, since the pattern would then track selective history rather than hierarchical position.

#### Prediction 3: bidirectional intergenerational consequences of architectural perturbation at germline-soma coupled components

This prediction concerns consequences across the germline-soma axis: the partition of an organism’s cells into those that contribute genetic and regulatory material to descendants (the germline) and those that do not (the soma), together with the documented signalling mechanisms by which information passes between them. Some regulatory components operate in parallel in both compartments without any documented mechanism connecting them: piRNA pathway components (Ross et al., 2014), chromatin-maintenance machinery (Allis and Jenuwein, 2016), and mobile-element surveillance (De Cecco et al., 2019; Gorbunova et al., 2021). The prediction below concerns only the narrower case where such a mechanism is documented (Kishimoto et al., 2017). ‘Intergenerational’ is used here for descendant-generation effects generally, and ‘transgenerational’ where effects appear in generations never directly exposed (Heard and Martienssen, 2014).

Perturbation of regulatory components coupled between germline and soma by a documented signalling mechanism should produce structured intergenerational consequences in both directions: from germline disruption to descendant somatic phenotype, and from somatic disruption affecting germline-active components to descendant consequences. The prediction is not that intergenerational effects are widespread (many perturbations should produce no descendant consequences), but that, where such effects occur, their distribution and character should be predictable from the regulatory role of the coupling mechanism connecting germline and somatic systems.

##### Discriminating force

Three features make the prediction discriminating against alternative accounts. First, the prediction commits to bidirectionality: intergenerational consequences should be detectable in both directions when coupled components are perturbed, with magnitude and form reflecting the regulatory role of the component in each domain; observation of consequences in only one direction is informative rather than disconfirming, provided the asymmetry is itself architecturally interpretable. Second, the prediction commits to structural specificity: effects should map onto the architectural role of the perturbed component (chromatin-dependent regulatory states for a chromatin-maintenance component; genomic stability for a mobile-element-surveillance component) rather than appearing as undifferentiated dysfunction. Third, and most importantly, the prediction commits to architectural selectivity, which must be stated carefully: the claim is not that a component being expressed in both germline and soma is sufficient for cross-generational effects, since parallel instantiation in two tissues is not itself a channel between them. The claim is the stronger one: perturbation should produce structured, direction-specific descendant consequences only where a signalling mechanism couples somatic and germline compartments, with their form predictable from the mechanism’s regulatory role. The clearest such case is the soma-to-germline circuit discussed below. Where no such mechanism exists, perturbation confined to soma should produce no descendant effect at all, consistent with the general restriction on soma-to-germline transfer recognised in the literature (Perez and Lehner, 2019). This is a strong falsifiable claim, since it commits in advance to which perturbations should and should not yield descendant effects on mechanistic grounds.

##### Postdictive evidence

The empirical record bearing on this prediction is thinner than for Predictions 1 and 2, and much of its discriminating force is prospective rather than postdictive. The clearest case, and the one that demonstrates a documented coupling mechanism rather than merely parallel instantiation of a shared component, is the DAF-16/FOXO- and HSF-1-dependent process in *C. elegans*, in which parental somatic tissue establishes an H3K4me3-based epigenetic memory in the germline, subsequently read out in descendant somatic tissue via SKN-1/Nrf to confer transgenerational stress resistance (Kishimoto et al., 2017). Further supporting evidence comes from work on RNA-induced epigenetic silencing in the same species, which has shown that perturbations to specific small-RNA pathway components produce structured transgenerational consequences whose form maps onto the regulatory role of the perturbed component. Such effects persist across multiple generations and are extinguished in characteristic patterns when the architectural component is restored (Rechavi et al., 2014; Houri-Ze’evi et al., 2016). Mammalian evidence for transgenerational epigenetic effects is more contested and harder to interpret architecturally, partly because *in utero* exposure cannot be cleanly separated from gametic transmission and partly because the structural specificity of reported effects has not generally been mapped onto a documented coupling mechanism of the kind identified in *Caenorhabditis elegans* (Heard and Martienssen, 2014). The prediction is consistent with this evidence where it is strongest, but it does not depend on the broad transgenerational-inheritance literature being correct in its current form. What it predicts is more specific: that *structured* intergenerational consequences should follow from perturbation of components with a documented coupling mechanism, in patterns that reflect the regulatory role of that mechanism, and that no such effects should follow where no coupling mechanism operates.

##### Comparison with alternative accounts

The architectural account differs here from two alternatives. A standard genetic-inheritance account (Futuyma and Kirkpatrick, 2022), in which heritability is essentially the transmission of DNA sequence and most epigenetic effects are dismissed as noise, predicts that perturbations leaving the sequence unaltered should produce descendant consequences that are absent or sporadic, since the regulatory states they disturb are reprogrammed each generation. The architectural account instead predicts they should be structured and predictable in distribution, character, and directionality from the regulatory role of the documented coupling mechanism. A second alternative is itself an auxiliary move relative to the standard account. The transgenerational epigenetic inheritance literature treats such effects as a distinct class of phenomena requiring their own explanatory apparatus, often through novel mechanisms specific to epigenetic transmission (Jablonka and Lamb, 2005). This position is contested within the broader literature on the Weismann barrier itself (Nilsson et al., 2020; see Heard and Martienssen, 2014 for a more cautious assessment). The architectural account does not deny such mechanisms but reframes them as the across-generation expression of the same organisational logic that operates within organisms: it rests not on epigenetic transmission in general but on documented coupling mechanisms between germline and somatic systems, predicting that disruption of such a mechanism produces consequences whose structure reflects its regulatory role.

##### Verdict criteria

Structured intergenerational consequences whose distribution maps onto the regulatory role of a documented coupling mechanism, in either direction, would support the prediction. Consequences appearing stochastically or confined to specific exposure types without such a mechanism would not discriminate, being compatible with accounts that treat such effects as noise. Systematic failure of components with a documented coupling mechanism to produce structured consequences, particularly in tractable systems such as *C. elegans*, would weigh against the prediction, which is testable through systematic experimental study of candidate coupling mechanisms. Structured, direction-specific consequences persisting where systematic investigation has failed to identify any coupling mechanism would weigh against the prediction’s selectivity claim; their appearance where none has yet been sought would instead identify one to look for.

### Prediction following from germline priority in propagation

Without the integrity of germline regulatory systems, the architecture cannot be transmitted, and what cannot be transmitted cannot sustain evolutionary coherence over deep time (the hypothesis’s second commitment; see Introduction). This is a stronger claim than the standard genetic-inheritance framework’s germline privilege, which rests only on the narrower ground that germline cells alone contribute to descendants. The architectural commitment holds that what the germline transmits includes regulatory architecture as architecture, not only DNA sequence and a limited set of chromatin states, but the regulatory relations through which each generation’s configuration is re-established, as in the sequence-independent propagation of centromere identity (Part I, Section 5). A specific empirical signature follows: disruption of germline regulatory architecture should propagate forward in a characteristic pattern, with lineages remaining reproductively viable for several generations before crossing a threshold beyond which transmission fails. The same logic extends to within-organism timescales, where components active in both germline and soma should show parallel failure patterns (across-generation erosion in the germline, within-organism cumulative dysregulation in soma) when compromised. Prediction 4 develops both directions.

Prediction 4’s somatic analogues rest on a weaker structural requirement than Prediction 3’s cross-domain claim: they do not require a documented signalling channel between germline and soma, only that the same architectural component, independently instantiated in each compartment, fail in structurally homologous ways when compromised in either, without either compartment’s failure causing the other’s.

#### Prediction 4: intergenerational erosion under partial germline compromise, with somatic analogues at shared architectural components

Partial disruption of germline regulatory architecture should produce a distinctive temporal signature, termed the Mortal Germline (MRT) pattern after its clearest empirical instance: lineages may remain reproductively viable across multiple generations while exhibiting cumulative degradation of regulatory coherence, eventually crossing a threshold beyond which transmission fails. Where the same architectural components are active in somatic tissues, their compromise should produce within-organism analogues of this across-generation erosion pattern, in which cumulative architectural degradation drives progressive somatic dysregulation rather than immediate failure.

##### Discriminating force

Three features make the prediction discriminating against alternative accounts. First, the predicted temporal signature is distinctive. Multi-generational viability followed by threshold-dependent collapse differs from both immediate sterility (predicted by accounts of severe component-level failure) and gradual decline (predicted by mutation-accumulation accounts). This is because what is being eroded is not a feature directly accessible to phenotypic selection within any single generation, but the architecture through which the conditions of viable transmission are re-established in each generation. Collapse is threshold-dependent rather than gradual since viable transmission is sustained across a range of architectural states and fails qualitatively at the envelope boundary (Part I, Section 6). Second, the prediction commits to recurrence across architectural components. The Mortal Germline pattern should appear at any architecturally central germline regulatory system, not be specific to any particular gene or pathway, as the empirical record already shows across the piRNA pathway, nuclear RNAi, heterochromatin maintenance, germ-granule integrity, and telomere maintenance, with the prediction extending to components yet to be characterised. Third, the prediction commits to somatic-germline structural homology: where components are shared between germline and soma, their compromise should produce parallel failure modes (across-generation erosion in the germline, within-organism cumulative dysregulation in soma), the somatic manifestation reproducing the germline case’s architectural signature rather than a separate cellular-failure mode. These are parallel expressions of one architectural fragility, not effects transmitted between compartments as in Prediction 3.

##### Postdictive evidence

The germline-direction prediction is well supported by an established but underintegrated body of evidence on the Mortal Germline (MRT) phenotype in *C. elegans*, not previously articulated, to our knowledge, as a unified consequence of a single architectural hypothesis. Mutations in the Piwi-related gene *prg-1* and other piRNA-pathway components do not produce immediate sterility (initial generations are reproductively viable) but a transgenerational decline in fertility over several generations, culminating in sterility at a characteristic threshold generation (Simon et al., 2014; Heestand et al., 2018). The phenotype is not specific to *prg-1*: a class of *mrt* mutations involving nuclear RNAi components, heterochromatin maintenance machinery, germ-granule integrity, and ribosomal RNA pool regulation all produce progressive multi-generational decline, with germ-granule dysfunction, perinuclear alteration, and reproductive arrest at the generation of sterility (Spracklin et al., 2017; Wahba et al., 2021). Loss of telomere maintenance produces a parallel but mechanistically distinct form of transgenerational sterility, exhibiting the same structural pattern of multi-generational viability followed by threshold-dependent collapse (Ahmed and Hodgkin, 2000). What unites these cases is the prediction’s central feature: partial compromise of germline regulatory architecture is compatible with immediate viability while cumulatively eroding the conditions of continued transmission, with collapse arriving when cumulative compromise crosses a threshold rather than as an immediate consequence of the initiating perturbation.

The somatic-direction prediction extends this logic: the same architectural fragility that manifests across generations in the MRT phenotype should manifest within organisms as cumulative somatic dysregulation, in patterns reflecting the compromised component’s architectural role. Observations of transposable-element derepression in somatic tissues driving age-related inflammation, cellular senescence, and a range of pathologies (De Cecco et al., 2019; Gorbunova et al., 2021; Della Valle et al., 2025) are consistent with this expectation: the loss of mobile-element surveillance that produces germline collapse across generations in the MRT phenotype produces, in somatic contexts, the cumulative inflammatory and senescence signatures that drive the somatic pathologies treated under Prediction 6. Loss of heterochromatin integrity in cancer, with associated transposable-element activation and genomic instability (Burns, 2017), exhibits the same architectural signature (unconstrained mobile-element activity following loss of the regulatory control that ordinarily contains it) that the germline MRT phenotype exhibits across generations. The somatic and germline manifestations are not analogies in the loose sense; they are the within-organism and across-generation expressions of the same architectural failure mode at the same regulatory components.

##### Comparison with alternative accounts

The architectural account predicts the multi-generational-viability-then-collapse signature as a structural consequence of how germline regulatory architecture is constituted, against two alternatives that do not predict it. A standard selectionist account does not predict this signature. While early generations remain reproductively normal there is no fitness differential on which selection can act, so the compromise stays invisible to it until collapse is already under way; and mutation-selection balance alone cannot explain MRT’s persistence in wild populations without invoking balancing selection (Saber et al., 2022). What the architectural account adds is why the compromise should be invisible in this way: what is eroded is the architecture sustaining the conditions of viable transmission, not a currently expressed fitness component. A mutation-accumulation account could in principle produce multi-generational decline of some kind, but would predict gradual rather than threshold-dependent collapse and would not specifically predict the constellation of architectural phenotypes (germ-granule dysfunction, perinuclear alteration, threshold-dependent reproductive arrest) the MRT literature documents. The architectural account predicts both the temporal signature and the constellation, since both follow from the structural role of the perturbed components in maintaining viable germline organisation.

##### Verdict criteria

Extension of the MRT pattern to additional germline regulatory components, in *C. elegans* or other organisms, would support the prediction, as would somatic compromise of architecturally shared components producing failure modes structurally homologous to the germline MRT phenotype. Compromise of architecturally central germline components producing immediate sterility rather than multi-generational erosion, or gradual rather than threshold-dependent decline without the characteristic architectural phenotypes, would weigh against it. Somatic compromise of components not shared with the germline producing effects of the same architectural form would not discriminate, being compatible with broader cellular-failure accounts making no architectural commitment.

### Prediction following from the coherence-breakdown relationship

The hypothesis’s third commitment (see Introduction) differs from the view that pathology involves additional pathological mechanisms imposed on an otherwise intact system. On the architectural account, entrenched pathological states arise without any additional apparatus: when upstream conditions (the inputs feeding a regulatory architecture) are displaced from their normal range, the same architectures that sustain coherent function come to sustain alternative dysregulated regimes that resist return to prior function. If this is correct, the feedback architectures sustaining a dysregulated regime should be the same architectures that, under different conditions, stabilise a coherent one. Prediction 5 develops this expectation.

#### Prediction 5**:** entrenchment of pathological regimes through the same feedback architectures that sustain coherent function

In entrenched pathological states, the feedback architectures sustaining the dysregulated regime should be the same architectures that, under different upstream conditions, stabilise a coherent functional regime. Entrenchment is therefore predicted to arise without any additional pathological apparatus imposed on top of an otherwise intact system: the architecture that stabilises the pathological state is the architecture that ordinarily stabilises function, repurposed under different inputs. Figure 2 illustrates this pathway from a healthy regime to entrenched pathology.

**FIGURE 2:**
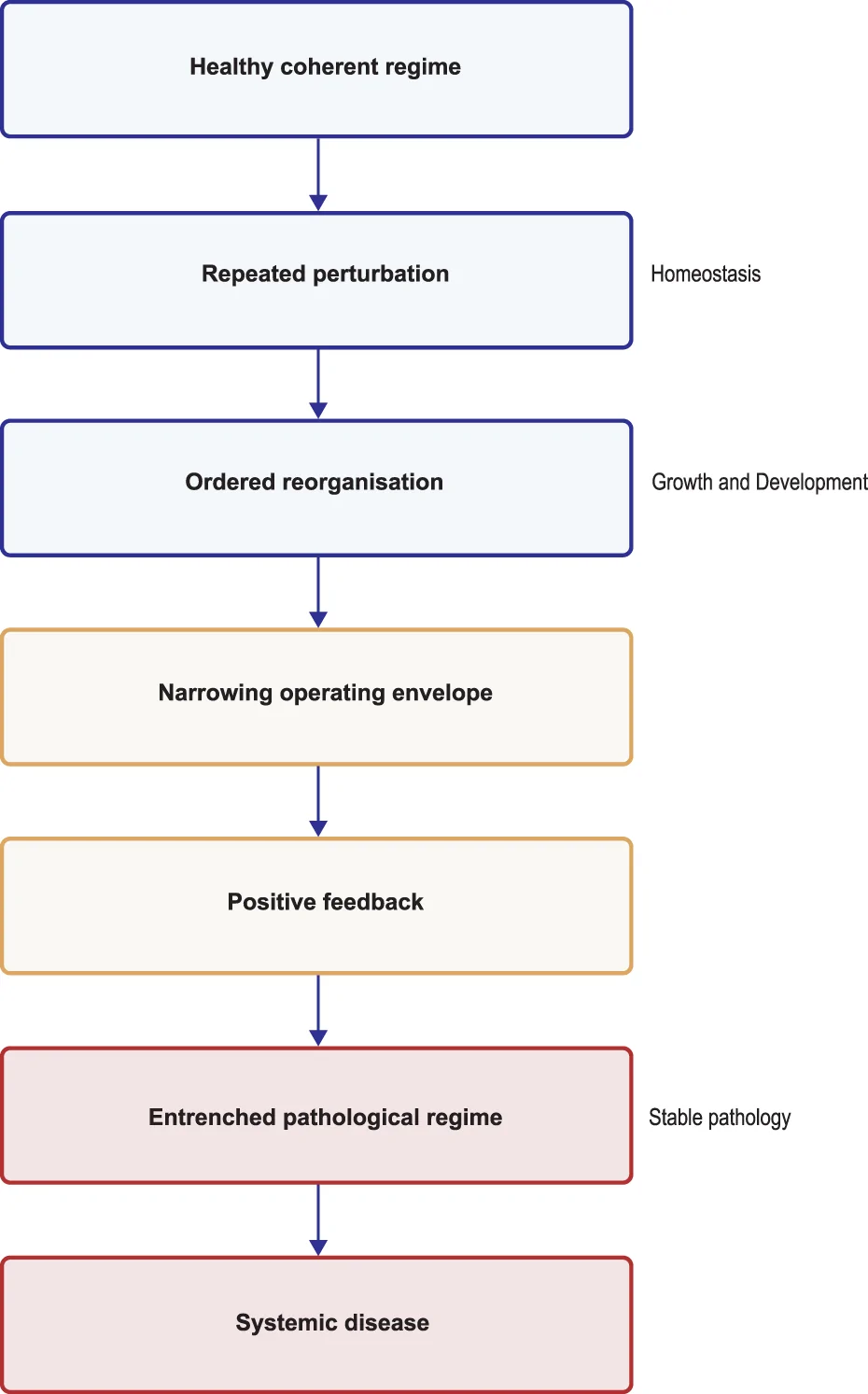
The pathway from a healthy coherent regime to entrenched systemic disease. Repeated perturbation is initially met by ordered reorganisation (Part I, Section 6), preserving coherence at a higher level. Where the operating envelope narrows, whether through accumulated perturbation, chronic stress, or age, the system becomes more susceptible to crossing this threshold; once displacement occurs, the same feedback architectures that stabilise coherent function are capable of stabilising the alternative regime instead (Part I, Section 6), producing an entrenched pathological regime (Prediction 5) that presents clinically as systemic disease. The side labels name the general regime each labelled stage instantiates: homeostasis (the maintenance of the healthy regime under repeated perturbation), growth and development (an organism-scale instance of ordered reorganisation; Part I, Section 2), and stable pathology.

##### Discriminating force

Four features make the prediction discriminating against alternative accounts. First, the prediction is not the generic observation that any regulated system admits multiple states. It is the more specific structural-homology claim that, in entrenched pathologies, the regulatory motif sustaining the pathological regime is the same motif whose normal role is to stabilise an alternative healthy regime, now operating under displaced upstream conditions. This requires that the cases examined under Postdictive evidence be instances of the same architecture in different regimes, not architectures that merely resemble one another. Second, the prediction is empirically demanding: healthy and pathological regimes should share not merely overlapping components but the same architectural role, operating under different upstream conditions. Third, the prediction commits to a regime-shift therapeutic logic. Effective intervention should generally require destabilising the pathological regime and re-establishing the upstream conditions for the healthy one, rather than removing a pathological component with no healthy counterpart. This is consistent with the repeated finding that component-targeting produces only partial or temporary effects in entrenched conditions compared with regime-shifting interventions (Holohan et al., 2013). Fourth, the prediction is prospectively extendable: given a regulatory architecture with bistable or multistable feedback, an entrenchable pathological regime should be predictable from the architecture itself, prior to clinical observation, specifying a structural feature sufficient to identify susceptibility before any pathology has occurred.

##### Postdictive evidence

The prediction is supported by a body of mechanistic evidence not previously articulated, to our knowledge, as a unified consequence of a single architectural hypothesis. In fibroblast biology, the same bistable positive-feedback loop maintaining differentiated fibroblast identity under normal conditions stabilises the persistently activated state characteristic of fibrosis and tumour stroma, with the upstream condition alone determining which state the switch occupies (Yeo et al., 2018). In chronic inflammation, the homeostatic feedbacks that normally resolve inflammatory responses come, when displaced sufficiently far, to sustain a chronic regime driving oncogenic and degenerative pathology: the same resolution machinery operating under different conditions, not a distinct pathological mechanism (Schwartsburd, 2003; Medzhitov, 2008). In epithelial-mesenchymal transition (EMT), the machinery driving normal developmental and wound-healing roles is the same machinery driving pathological EMT in metastatic progression, the difference lying in whether the regulatory context bounds it or allows it to become sustained and progressive (Nieto et al., 2016). In therapeutic resistance, compensatory reactivation of survival pathways following targeted inhibition exploits the same redundancy architectures that support cellular robustness under normal conditions: the redundancy that makes normal cells robust is what makes pathological cells resistant (Holohan et al., 2013).

##### Comparison with alternative accounts

The architectural account predicts this structural relation as a general property of regulatory systems whose feedback architectures support multiple stable regimes, against two alternatives. The view that pathology involves additional pathological mechanisms imposed on an otherwise intact system would predict that entrenched states require pathological apparatus distinct from anything operating under healthy conditions: specific oncogenic feedbacks, autoimmune amplification loops, or fibrotic activation circuits with no normal counterpart. A related assumption in oncology, that therapeutic resistance reflects the selection of mutant clones rather than reversible, non-genetic states drawing on normal cellular machinery, has itself recently been named as a prevailing dogma and contested (Kulkarni et al., 2023). The mechanistic record reviewed above is hard to reconcile with this: the feedbacks sustaining entrenched pathology are repeatedly found to be the same feedbacks sustaining healthy function, not additional ones. A component-level account, in which entrenchment reflects the persistence of pathological component states, makes no specific prediction about structural homology between healthy and pathological feedback use, and is compatible with either the observed pattern or its absence.

##### Verdict criteria

Further entrenched pathological states sustained by feedback architectures whose normal role is to stabilise healthy regimes would extend the postdictive support, as would regime-shift therapeutic interventions proving systematically more durable than component-targeting ones in entrenched conditions. Entrenched states sustained by feedback architectures with no healthy counterpart, or bistable architectures whose pathological-regime susceptibility cannot be predicted from the architecture itself, would weigh against the prediction. Entrenchment reducible to the persistence of a particular component state without feedback-architecture involvement would not discriminate, being compatible with component-level alternatives making no commitment about feedback structure.

### Prediction following from disruption at the organism level and the convergent signatures of systemic disease

Distinct chronic conditions converge on shared signatures (see Introduction), the same architectures, conserved over evolutionary time, fail in characteristic ways determined by their structure, so that distinct chronic conditions converge on shared signatures because what fails has the same architectural shape even where the molecular substrate differs. Prediction 6 develops this expectation, identifying the specific class of conditions to which it applies and the signatures the convergence is predicted to take.

#### Prediction 6: convergent architectural signatures across distinct systemic pathologies

Conditions characterised by distributed regulatory dysregulation (including many cancers, chronic inflammatory and autoimmune conditions, neurodegenerative diseases, and age-related decline) should converge on shared signatures of architectural breakdown. Three such signatures follow from the framework. The first is loss of hierarchical regulatory constraint, in which deeply embedded regulatory components disengage from the more peripheral systems they ordinarily constrain. The second is inappropriate activation of context-restricted regulatory states, in which developmental, embryonic, or otherwise context-restricted regulatory states are expressed in tissues or contexts where they are normally suppressed. The third is the emergence of self-sustaining dysregulated regimes, in which feedback architectures that under normal conditions return the system toward its operating envelope come instead to stabilise a displaced regime in which dysregulation is maintained rather than corrected.

##### Discriminating force

Three features make the prediction discriminating against alternative accounts. First, the prediction commits to convergence across a specific class of pathologies, those involving distributed regulatory dysregulation rather than localised lesions; component-level accounts make no specific prediction about which classes should converge, whereas the architectural account predicts convergence precisely where what fails is architecture rather than individual components. Second, convergence should take particular forms rather than being arbitrary: the three named signatures should recur across the class, not some other dimension unrelated to architectural breakdown. Third, and most strongly, convergence should not be reducible to shared molecular components: it should appear even where the underlying machinery differs across pathologies, because what is shared is the mode of architectural failure rather than the substrate, whereas a component-level account predicts shared signatures only where components are literally shared.

##### Postdictive evidence

The catalogued character of disease is itself an explanandum the architectural account organises: pathology resolves into recognisable, recurrent forms across the descriptive medical tradition (organ-system, histological pattern, syndromic constellation, natural history) rather than appearing as an unstructured field of dysfunction, and each of these axes is itself architectural rather than component-aggregative. An organ system, for instance, is a functional architecture identified by the role its parts jointly perform, not merely an anatomical region, a status the cardiovascular system attained only after Harvey’s account of circulation integrated components whose participation in a single circulatory architecture had not previously been recognised (Harvey, 1628). A purely component-level account does not predict that the relevant units of disease cataloguing should be functional architectures rather than molecular components; the architectural account predicts this because what fails in the pathologies under consideration is the architecture, not the components in isolation. What the architectural account contributes is a framework within which these axes are intelligible as expressions of the same organisational logic Part I identified at developmental, germline, chromatin, and metabolic scales, and within which the convergent failure modes documented in modern molecular biology are predicted rather than coincidental.

The convergent findings of modern molecular biology illustrate the first two signatures under more fine-grained analysis. The hallmarks-of-cancer framework (Hanahan and Weinberg, 2011; Hanahan, 2022) and the hallmarks-of-aging framework (López-Otín et al., 2013) identify substantially overlapping signatures—genomic instability, epigenetic alterations, deregulated nutrient sensing, cellular senescence, altered intercellular communication, and loss of proteostasis—each reflecting either the loss of hierarchical constraint over components normally kept in check, or the inappropriate activation of regulatory states normally restricted to development. Integrative work has subsequently extended these overlaps to immune dysfunction and to neurodegeneration (Chen et al., 2025). Cancer and neurodegeneration, despite operating in opposite directions at the cellular level (proliferative versus degenerative), are reported to share dysregulation of cell-cycle machinery, redox homeostasis, DNA-damage response, and inflammatory signalling. Network-medicine analyses point in the same direction from a different angle: disease modules in the human interactome (the network of protein and gene interactions in human cells) are not isolated, but partially overlap, with a small number of shared modules implicated across many otherwise distinct pathologies (Goh et al., 2007; Suthram et al., 2010; Barabási et al., 2011; Menche et al., 2015).

The third signature (entrenchment through feedback architectures that under normal conditions sustain coherent function) is treated in its own right in Prediction 5, where the structural relation between feedback that sustains normal function and feedback that sustains pathology is examined directly. Within the present prediction, it appears as one of three convergent signatures. The same structural relation recurs across otherwise distinct pathologies: chronic inflammation as a self-sustaining loop driving pro-cancer microenvironments (Schwartsburd, 2003); bistable feedback producing perpetual fibroblast activation in fibrotic and tumour-stromal states (Yeo et al., 2018); and mechanosensory feedback sustaining chronic inflammatory and fibrotic conditions (Saha et al., 2023). Therapeutic resistance in cancer, in which inhibition of one survival pathway is followed by compensatory reactivation of another (Holohan et al., 2013), exhibits the same structural pattern at the level of clinical intervention.

##### Comparison with alternative accounts

The architectural account predicts these convergences as expected; standard alternative engagements do not. A purely component-level account treats each pathology as the malfunction of distinct molecular components and predicts shared signatures only by coincidence of component overlap, not as a structural property of how regulatory architectures fail. Network medicine and the hallmarks frameworks document these convergences empirically, but as auxiliaries to a component-level framework, establishing that the convergence is real while leaving implicit the architectural level that would explain it. The architectural account makes that level primary, predicting convergence as a structural consequence of architectural failure rather than an empirical regularity to be documented.

##### Verdict criteria

Confirmed convergence on these particular signatures, across pathologies with otherwise dissimilar mechanistic profiles, would support the architectural account. Convergence reducible entirely to shared components would weigh against the prediction’s strongest claim, that convergence appears even where the underlying machinery differs, while remaining compatible with component-level accounts. Failure of this class of pathologies to exhibit such convergence would weigh against the prediction.

Table 1 summarises the six predictions, identifying for each the architectural commitment from which it follows, the postdictive evidence it organises, the discriminating claim that distinguishes it from alternative accounts, and its current empirical status. The testability and evidential standing of the six predictions, taken together, are examined in the Discussion. Section 2 examines the implications of this framework for systemic health, several of which the predictions developed above already make visible.

**TABLE 1:** Summary of the six predictions developed in Section 1. Each prediction is shown with the architectural commitment from which it follows, the postdictive evidence it organises, the discriminating claim that distinguishes it from alternative accounts, and its current empirical status. GRN, gene regulatory network; EMT, epithelial-mesenchymal transition.

Prediction	Architectural commitment	Postdictive anchor	Discriminating claim	Status
1. Catastrophic failure at architecturally central components	Integrated operation across cells, tissues, organisms, and generations	Catastrophic failure of GRN kernels (Davidson and Erwin, 2006); cascading germline collapse following piRNA pathway disruption (Malone and Hannon, 2009); chromosome mis-segregation following CENP-A loss without graded intermediates (McKinley and Cheeseman, 2016)	Catastrophic failure is concentrated at architecturally central nodes and tracks centrality rather than the strength of selection on phenotypic outputs	Postdictive
2. Mid-hierarchical concentration of lineage transitions	Integrated operation across cells, tissues, organisms, and generations	GRN sub-circuits as locus of body-plan reorganisation (Davidson and Erwin, 2006; Erwin and Davidson, 2009); cis-regulatory evolution at intermediate components (Carroll, 2008; Wittkopp and Kalay, 2012)	Lineage transitions are non-randomly concentrated at mid-hierarchical regulatory levels, with the concentration holding across clades whose ecological pressures differ substantially	Mixed
3. Bidirectional intergenerational consequences at coupled components	Integrated operation across cells, tissues, organisms, and generations	Documented soma-to-germline-to-soma signalling circuit conferring transgenerational stress resistance (Kishimoto et al., 2017); structured small-RNA-mediated transgenerational effects in *C. elegans* (Rechavi et al., 2014; Houri-Ze’evi et al., 2016); cautious interpretation of mammalian transgenerational evidence (Heard and Martienssen, 2014)	Intergenerational consequences depend on a documented coupling mechanism between germline and soma, not merely on parallel instantiation of a shared component; only components with such a mechanism produce structured bidirectional effects, with form predictable from the mechanism’s regulatory role	Mainly prospective
4. Intergenerational erosion with somatic analogues	Germline priority in propagation	Mortal Germline phenotype across piRNA, nuclear RNAi, heterochromatin, germ-granule, and telomere-maintenance systems (Ahmed and Hodgkin, 2000; Simon et al., 2014; Spracklin et al., 2017; Heestand et al., 2018); somatic mobile-element derepression and age-related dysregulation (De Cecco et al., 2019; Gorbunova et al., 2021)	Multi-generational reproductive viability followed by threshold-dependent collapse, with within-organism analogues at architecturally shared somatic components	Postdictive (germline); mixed (soma)
5. Entrenchment via shared feedback architectures	Coherence-breakdown relationship	Bistable fibroblast feedback in fibrosis and tumour stroma (Yeo et al., 2018); chronic inflammation as homeostatic feedback in pathological regime (Schwartsburd, 2003; Medzhitov, 2008); pathological EMT using developmental machinery (Nieto et al., 2016); compensatory pathway reactivation in resistance (Holohan et al., 2013)	The feedback sustaining entrenched pathology is the same regulatory architecture that sustains a healthy regime under different upstream conditions, not an additional pathological mechanism	Postdictive
6. Convergent architectural signatures across pathologies	Disruption at organism level and convergent signatures of systemic disease	Overlap of hallmarks across cancer, ageing, immune dysfunction, and neurodegeneration (Hanahan and Weinberg, 2011; López-Otín et al., 2013; Chen et al., 2025); disease-module overlap in the human interactome (Goh et al., 2007; Menche et al., 2015)	Distinct pathologies converge on three named architectural signatures even where the underlying molecular machinery differs, because what is shared is the mode of architectural breakdown rather than the molecular substrate	Postdictive

## Implications for systemic health

Each of the hypothesis’s four commitments bears on systemic health in its own way. The architecture’s integrated operation across the two scales of evolutionary and within-organism coherence underlies the bidirectional intergenerational consequences of perturbation at germline-soma coupled components (Prediction 3). Germline priority in propagation underlies the somatic analogues of germline dysregulation at shared architectural components (Prediction 4). The coherence-breakdown relationship underlies the entrenchment of pathological regimes through the same feedback architectures that sustain coherent function under different conditions (Prediction 5). Disruption of the architecture at the organism level underlies the convergence of distinct systemic pathologies on shared architectural signatures (Prediction 6). The present section works through what these connections imply for how systemic health is conceptualised: not as a downstream application of the framework’s case for evolutionary coherence alone, which Part I calls its principal concern, but as an equally fundamental expression of within-organism coherence. Findings at either scale therefore bear on the framework’s standing in the other. It does not attempt a medical account of any particular condition, nor does it replace the mechanistic work required to understand specific pathologies. On this understanding, systemic health is the within-organism instantiation of architectures whose conservation across deep evolutionary time sustains coherent biological function.

## Health and disease as alternative regimes of the same architecture

On the present hypothesis, the architectures sustaining biological coherence also underlie the characteristic modes of its failure when their within-organism instantiation is disrupted. This bears most directly on entrenched systemic disease — cancer, certain autoimmune conditions, some neurodegenerative and metabolic conditions, and age-related decline—rather than on disease in general. For these conditions, disease is neither an external insult to an otherwise healthy system, nor reducible to the malfunction of an isolated component within an otherwise intact system; both framings treat it as imposed upon biological organisation from outside. The present hypothesis treats it differently: disease of this kind is a displacement of these same architectures, in their within-organism instantiation, into an alternative regime, made possible by the same regulatory capacities that stabilise the system under normal conditions. An infection, for instance, may still be usefully understood as an external insult in this sense, even if host susceptibility and the trajectory of the response it provokes are themselves architecturally mediated—a possibility this paper does not develop.

This reframing has two immediate consequences. First, the sharp ontological distinction between health and disease is softened. They are not organisational opposites, but alternative regimes of the same within-organism architecture. Second, understanding disease requires attention to the architecture that makes coherent function possible, not only to the mechanisms through which it fails. A purely proximate account may identify what has gone wrong in molecular terms, but cannot on its own explain why failure takes the forms it does, why some breakdowns stabilise, or why particular combinations of dysregulation recur across conditions that otherwise appear unrelated.

## Transient dysregulation, ordered reorganisation, and entrenchment

The threefold distinction developed in Part I, Section 6 (transient displacement, ordered reorganisation, and maladaptive reorganisation) maps directly onto health-relevant phenomena. Transient dysregulation is the familiar case: acute stress responses, reversible inflammation, and short-lived dysbiosis, in which the system is displaced and then returns. Ordered reorganisation is equally familiar, though less often framed in these terms: growth and development, puberty, pregnancy, wound healing, immune memory formation, and major physiological transitions, in which coherence is preserved at a higher level even as substantial reorganisation occurs, with the organism’s overall identity and function persisting across a change to its internal regulatory configuration.

The category the hypothesis renders especially salient is the third: maladaptive reorganisation that becomes entrenched, in which the system crosses into a dysregulated regime that stabilises rather than self-corrects. An architecture whose operating envelope has narrowed, whether through accumulated perturbation, chronic stress, or age, is more susceptible to crossing this threshold in the first place; once displacement occurs, the same feedback architectures that stabilise coherent function are capable of stabilising the alternative regime instead (Part I, Section 6). Figure 2 illustrates this pathway, from a healthy coherent regime through repeated perturbation and ordered reorganisation, to the narrowing of the operating envelope and the positive feedback that together produce entrenchment. As Prediction 5 develops in detail, this pattern recurs across cancer, certain autoimmune conditions, entrenched metabolic states, and some neurodegenerative and psychiatric conditions, despite major differences in proximate mechanism. This has a direct implication for how intervention is conceptualised: if entrenched pathology is sustained by the same architecture as healthy function, effective therapy cannot always be understood as correction of a component-level defect, and may instead require destabilising the pathological regime and re-establishing the upstream conditions for the healthy one, alongside or instead of addressing the initiating disturbance.

This regime-shift logic is visible in several areas of clinical practice, even where the architectural framing is not made explicit. Combination targeted therapy in oncology, in which simultaneous inhibition of multiple architecturally connected pathways produces more durable responses than sequential single-agent approaches, exemplifies it directly: single-agent inhibition is repeatedly followed by compensatory reactivation through redundancy architectures (Holohan et al., 2013), whereas combination approaches that engage the architecture sustaining the regime tend to produce more durable outcomes. In autoimmune conditions, sustained immunosuppression holds the system in a controlled state without restoring the architecture sustaining healthy tolerance, while tolerance-induction approaches pursue a genuine regime shift back toward healthy regulatory function; the former tends to require indefinite continuation, the latter aims at durable resolution (Junt et al., 2025). The architectural account does not specify which interventions will produce regime shifts in any particular condition, which remains the work of mechanistic clinical investigation; what it specifies is the structural logic effective intervention in entrenched pathology must satisfy.

## Developmental origins and ageing: coherence across the life course

Developmental robustness (the capacity to produce reproducible form despite perturbation) and the gradual decline of function in ageing are, on the present account, expressions of the same architectural integrity at different points in the life course. The GRN kernels conserved across the bilaterian body plan, the regulatory systems governing germline integrity, and the chromatin architectures sustaining cell identity are substrates of biological coherence whose within-organism expression is what developmental robustness reflects, and whose progressive erosion is what ageing tracks. Disruption during development is therefore disruption to architectures whose integrity also sustains coherent function across the rest of the life course, predisposing the organism to characteristic forms of later dysfunction. The same architectures, absent any discrete disrupting event, undergo gradual degradation through ordinary use. This is what the present account offers as a more specific claim than generic maintenance-failure descriptions of ageing: not merely that maintenance fails, but that what degrades are the same architectures whose lineage-scale conservation sustains evolutionary coherence.

This reframing brings into focus two bodies of work often treated as separate empirical literature rather than expressions of a common principle. The Developmental Origins of Health and Disease framework, originating with Barker’s observations linking early-life conditions to adult disease (Barker, 1992) and subsequently developed through extensive empirical work (Gluckman et al., 2008), documents precisely the pattern the present hypothesis predicts. Early perturbation produces structured and recurrent effects on later susceptibility because what is perturbed is the within-organism operation of conserved architectures. At the other end of the life course, genomic and epigenetic instability (often described in component terms as mutation accumulation or loss of regulatory marks) is better understood as failure of the same maintenance systems. Evidence that such instability follows structured patterns across conditions (Gorbunova et al., 2021; López-Otín et al., 2023) is consistent with this interpretation. Prediction 4’s somatic-direction claim, that compromise of architecturally shared components produces within-organism analogues of the across-generation Mortal Germline pattern, locates this structured decline within the same architectural logic that operates in the germline.

As architectural integrity declines across the life course, the likelihood of maladaptive transitions increases, recovery becomes less probable, and multiple forms of dysregulation begin to converge. The familiar late-life co-occurrence of seemingly distinct conditions (immune dysfunction, metabolic dysregulation, neurodegeneration, and cancer) reflects this shared process of architectural degradation, and the convergent architectural signatures developed in Prediction 6 are intelligible as its expression across pathologies whose proximate mechanisms differ.

## Intergenerational dimensions of health

Because the CHORA Hypothesis assigns the germline a central role in the propagation of regulatory architecture, it brings intergenerational dimensions of health into closer relation with somatic health: the germline that transmits regulatory architecture from one generation’s instantiation to the next is the same germline whose conservation across deep time is what makes evolutionary coherence possible. Health in one generation depends in part on the integrity of the architecture inherited from previous generations, and health in subsequent generations depends in part on what is transmitted onward.

Disruptions to germline architecture should therefore have consequences in descendants, though the strength of that claim depends on whether a documented coupling mechanism is involved. Several lines of human evidence are consistent with such effects, including altered metabolic and cardiovascular phenotypes in descendants of the Dutch Hunger winter (Heijmans et al., 2008), sex-specific intergenerational effects in the Överkalix cohort (Pembrey et al., 2006), and altered glucocorticoid regulation in children of Holocaust survivors (Yehuda et al., 2016). These should be weighted differently from the *C. elegans* evidence discussed under Prediction 3. There, a documented mechanism connects a specific somatic signal to a specific germline chromatin state (Kishimoto et al., 2017), whereas in the human cohorts the mechanistic specificity and the strictly transgenerational character of each finding remain contested, and *in utero* exposure cannot be cleanly separated from direct gametic transmission (Heard and Martienssen, 2014). The present hypothesis is consistent with the human evidence where it is strongest, but its discriminating claims rest on the mechanistically documented cases; the human cohort evidence is offered as suggestive rather than as direct support. Rather than treating documented cases as exceptional, the present hypothesis predicts that, where a coupling mechanism exists, its effects should be structured, recurrent, and predictable from the mechanism’s regulatory role, as Prediction 3 develops.

## Architectural and adaptive framings of disease vulnerability

Evolutionary medicine has illuminated many aspects of disease vulnerability through adaptive explanations: mismatch (a poor fit between physiology shaped by past selective environments and present conditions) (Gluckman and Hanson, 2006), trade-offs between competing fitness demands, and antagonistic pleiotropy (genes beneficial early in life becoming detrimental later) (Williams, 1957). These accounts operate within a selection-centred framework, explaining why vulnerabilities persist in populations.

The present hypothesis offers a complementary perspective: rather than asking only why vulnerability has been retained, it asks how the architectures sustaining biological coherence render organisms susceptible to particular forms of breakdown. Entrenchment, recurrent instability, developmental continuity, and intergenerational transmission are more readily addressed in architectural than adaptive terms.

The two approaches are complements, not competitors: adaptive accounts address why vulnerabilities persist in populations over evolutionary time, architectural accounts address both how and why they take the specific forms they do, and a complete understanding of disease likely requires both.

## Discussion

### The ontological status of CHORA

Part I characterises CHORA functionally, as an architecture whose integrity sustains biological coherence, defined by an operating envelope, boundary dynamics, and a mode of propagation across generations (Part I, Section 6). Two further aspects of this characterisation matter here. First, the architecture is common to the organism and the lineage, in the sense of one organisational logic sustaining both within-organism and evolutionary coherence (Part I, Section 7). Second, it is “higher-order,” meaning that it is neither reducible to the five molecular substrates nor an additional substrate amongst them, but the level at which their joint operation is organised. Figure 3 illustrates this relation between the five molecular substrates and the architecture they jointly realise.

**FIGURE 3:**
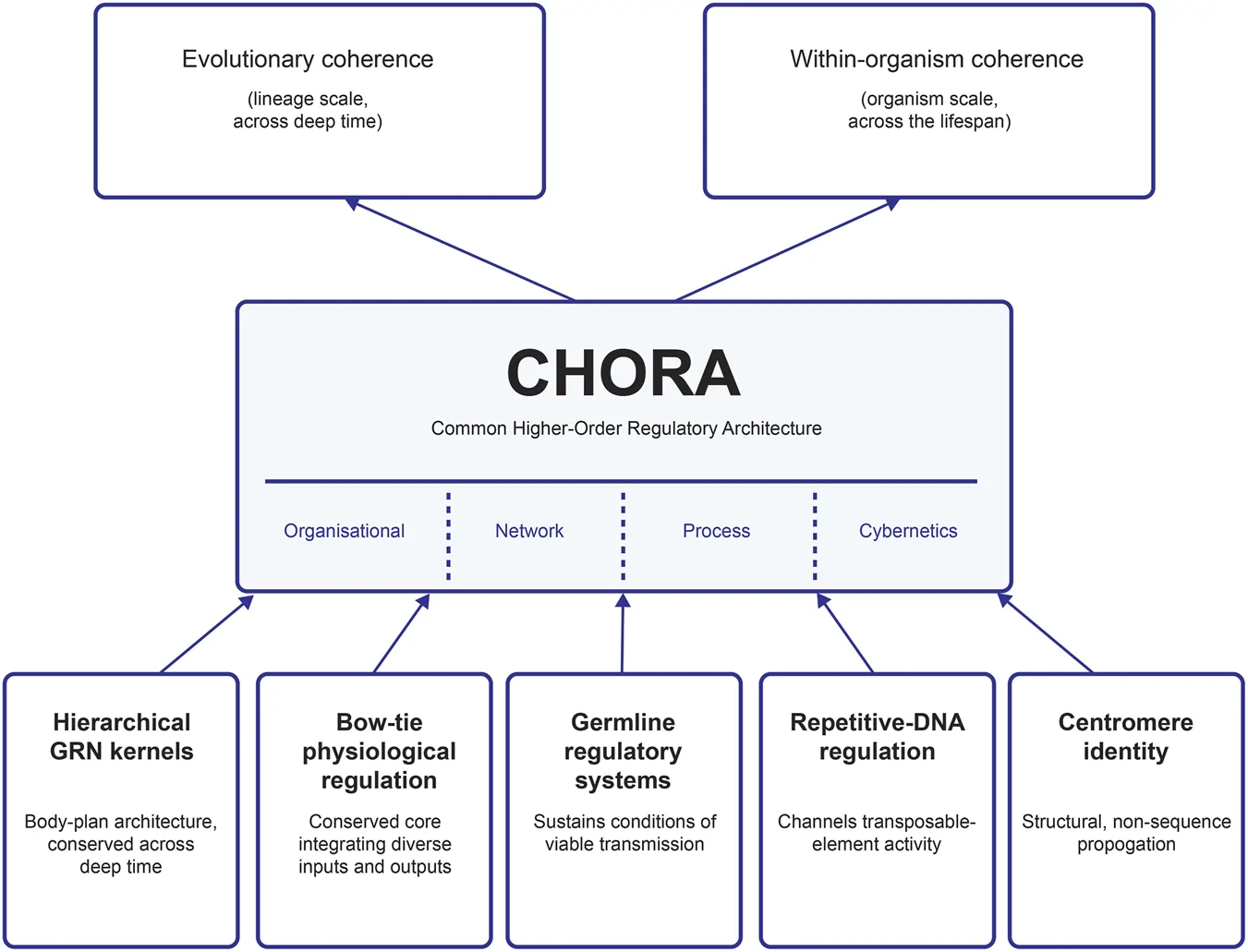
The CHORA architecture and its five molecular instantiations. The five molecular architectures examined in Part I, Section 5 (hierarchical GRN kernels, bow-tie physiological regulation, germline regulatory systems, repetitive-DNA regulation, and centromere identity) jointly realise CHORA, the organisational level at which their operation is integrated; CHORA is not a sixth substrate alongside them, nor a physical or formal construct independent of them. CHORA in turn sustains the two scales examined throughout this series: evolutionary coherence at the lineage scale and within-organism coherence at the organism scale. The four properties shown (organisational, network, process, and cybernetic) are the descriptions developed in the Discussion, each tied to a measurable claim already at work in Section 1: organisational character to the catastrophic-versus-graded failure distinction in Prediction 1, network character to architectural centrality in Predictions 1 and 2, process character to the operating envelope and regime-shift logic of Prediction 5, and cybernetic character to the width of the operating envelope as it narrows with age, described in Section 2.

Part I did not settle what kind of thing this architecture is, because it was not part of its remit. Its task was simply to establish that it exists and does the explanatory work claimed for it. The ontological question becomes answerable only once the architecture’s discriminating predictions are in view, since those predictions, developed in this paper, give the characterisation empirical content rather than leaving it as stipulation.

Four such properties can be identified, each already established elsewhere in this paper rather than newly proposed, and each measurable in principle from claims made there.

It has an organisational character (Moreno and Mossio, 2015), maintaining itself through the joint operation of its parts rather than through any one component in isolation. This is what the catastrophic-versus-graded failure distinction in Prediction 1 already tests empirically: removal of a central component collapses the architecture’s capacity to sustain itself, rather than merely reducing its output.

It has a network character, realised through the five architectures surveyed in Part I, Section 5 (Barabási et al., 2011). Predictions 1 and 2 already measure this as architectural centrality: a component’s structural position and indispensability within the regulatory hierarchy.

It has a process character (Scheffer et al., 2001). The language of regimes used throughout this paper, health and disease as alternative regimes, entrenchment as a shift between them, treats CHORA as a stabilised process rather than a static structure. This is measurable as the dimensions of the operating envelope and the degree of displacement needed to cross into a different regime, the empirical content of Part I’s Figure 1, of Figure 2, and of Prediction 5.

And it has a cybernetic character, in Ashby’s (1956) sense that a regulator requires sufficient variety to counter the variety of disturbances it faces. The width of the operating envelope is an empirical proxy for this capacity: wider where the architecture can absorb perturbation, narrower where accumulated compromise has eroded it, as Section 2’s treatment of ageing describes.

What CHORA is not follows from the same characterisation: not a physical substance additional to the molecules that realise it, and not a formal or informational construct independent of any material system, since it does no explanatory work as an abstract pattern detached from the molecules that instantiate it.

None of this amounts to a single scalar measure of how much architecture a system possesses, a gap acknowledged in the Limitations below; what the four properties show is that measurement is distributed across Section 1’s discriminating claims rather than unified into one operational definition. The deeper ontological question of what kind of thing organisation of this sort ultimately is remains reserved for a separate forthcoming companion paper on the framework’s philosophical foundations.

### Relation to existing frameworks and literature

One might ask whether the six predictions merely restate well-established observations. But the distinguishing claim is not a new way of categorising these observations, nor a restatement of the systems-biology insight that robust architectures fail in characteristic ways (Kitano, 2004). It is the more specific claim that one conserved regulatory architecture is the shared substrate: that what is conserved across the lineage and what fails in disease are the same thing. Kitano treats the recurring architecture as a design motif reached repeatedly by convergent evolution, drawing on evidence that circuits arise independently rather than by duplication (Conant and Wagner, 2003) and that complete motifs are not propagated as units (Teichmann and Babu, 2004). That a circuit recurs across contexts is not evidence that it is independently reinvented on each occasion. In the developmental networks characterised by Erwin and Davidson (2009), the recurring subcircuits (their plug-ins) are conserved modules whose internal structure is invariant across deployments; what varies is where they are deployed, and deployment is governed by inherited cis-regulatory control circuitry. The architecture identified here as propagated is the higher-order regulatory architecture that Part I, Section 5 develops. Its paradigm instance is the recursively wired specification cores (kernels), which these authors distinguish from labile, redeployed subcircuits and whose disruption abolishes the structures they specify. Erwin and Davidson themselves conclude that the hierarchical network structure controls the nature of the variation available, packaging subcircuits for selection rather than assembling them anew. Convergence in where circuits are deployed is thus compatible with, and may be channelled by, conservation of the higher-order architecture that constrains which solutions are reachable. It yields assertions the standard account does not make and that could prove false, several prospective rather than postdictive; a recategorisation of known facts could not. It also achieves this with fewer independent assumptions. Where the standard frameworks append a distinct auxiliary hypothesis for each pattern selection did not predict, a single architecture that produces the patterns leaves those auxiliaries with no work to do. Selection itself is not displaced but operating within this architecture.

The six predictions have each been set against the relevant alternative account in Section 1, prediction by prediction. Taken together, those comparisons share one structure. In each case the standard framework leaves a variable to be settled after the fact by lineage-specific history—conservation by the strength of selection on outputs, intergenerational consequence by whatever transmits besides DNA sequence, pathological convergence by whatever components happen to be shared—and accommodates the observed pattern only by adding a hypothesis specific to it. The architectural account fixes the same variable in advance, from one commitment: conservation tracks architectural centrality independently of output-level selection (Predictions 1 and 2); descendant consequence follows a documented germline-soma coupling rather than parallel instantiation (Predictions 3 and 4); and pathological convergence reflects a shared mode of architectural breakdown rather than a shared molecular substrate (Predictions 5 and 6). Each is a dissociation the standard account leaves unspecified and CHORA derives structurally. This is the discriminating force meant throughout Section 1, and the respect in which the contribution is more than a recategorisation: not six separate observations differently labelled, but six consequences of one architectural commitment.

A related question is what the account adds to the broader traditions of organisational thinking, which Part I noted but did not develop. Cybernetics, organisational-autonomy theory, and the Extended Evolutionary Synthesis each commit, in general terms, to something like an architectural level shaping biological outcomes; what distinguishes the present account is not the idea, which they anticipate, but its conversion into six specific, falsifiable predictions.

From cybernetics, the account draws on the principle that a regulator must possess sufficient internal variety to counter the diversity of disturbances it faces (Ashby, 1956): the operating envelope in Part I, Section 6 is, in this sense, a claim about the regulatory capacity needed to absorb perturbation without loss of coherence. The account differs from classical cybernetics in its explanandum: Ashby’s framework addresses regulation and control generally, across biological and mechanical systems alike, and does not itself predict that catastrophic failure should track architectural centrality independently of selection strength on outputs (Prediction 1), a claim specific to this architecture rather than to regulated systems in general.

From organisational and autonomy-based approaches to biological systems (Moreno and Mossio, 2015), the account draws its central commitment: that biological systems are properly understood at the level of self-maintaining organisation, not only their component parts. The account extends organisational-autonomy theory in one direction: applying its reasoning jointly to lineage-scale persistence and within-organism operation, which the tradition has not generally addressed together, and from which Predictions 3 and 4’s claims about coupled and parallel germline-soma failure follow—claims organisational-autonomy theory in its existing form does not itself generate.

From the Extended Evolutionary Synthesis (Laland et al., 2015), the account draws its recognition that developmental processes, regulatory architecture, and inclusive inheritance structure the outcomes of selection rather than merely supplying the variation selection acts on. The account differs from the EES in taking the architectural level as primary and explanatory in its own right, rather than one factor among several alongside selection. This licenses Prediction 2’s stronger claim, that mid-hierarchical concentration of lineage transitions should hold across clades regardless of differing selective regimes, rather than the EES’s more general expectation that developmental structure will shape variation to some degree.

The architectural account is best understood, then, not as a departure from these traditions but as a synthesis that takes a claim each makes in part: regulatory sufficiency from cybernetics, self-maintaining organisation from autonomy theory, and structured variation from the EES. It applies these jointly to the two scales, lineage and organism, that the present series treats as expressions of a single architecture.

### Testability

Each of the six predictions carries its own explicit verdict criteria, set out in Section 1, specifying the observations that would extend, fail to discriminate, or weigh against it. Two features of the set as a whole bear on its standing.

First, the predictions differ in their present evidential standing, from substantially postdictive to mainly prospective; Table 1 summarises where each stands and what outstanding test its verdict criteria call for. The postdictive claims organise patterns individually well established but not previously unified. This unification is not the same as prospective confirmation, but neither is it mere redescription. It is the explanatory virtue on which the abductive case rests, and it accomplishes work that no component-level or selection-centred account is in a position to do, the drawing-together of independently documented patterns as consequences of one architecture.

Second, each prediction specifies in advance, via its verdict criteria, what would count against it, not only what would support it. The outstanding gaps are testable with current methods, and each would, if it failed rather than succeeded, count against the corresponding prediction rather than being absorbed as a further postdictive case.

### Limitations

The preceding sections have argued for the explanatory and discriminating value of the CHORA Hypothesis; this section sets out where the account is weakest, so that its adequacy can be assessed against its limitations as well as its successes.

#### Speculative aspects

The hypothesis is advanced by abductive inference, offered as the best available explanation of the patterns surveyed in Part I and the predictions developed here, not as a conclusion established by direct demonstration. This is appropriate to a Hypothesis and Theory article, but means the unifying account could in principle be replaced by a different one not yet articulated, a possibility the postdictive support in Section 1 cannot rule out. The claim that the five molecular architectures in Part I are expressions of a single organisational level, rather than five independently evolved solutions to similar problems, is the hypothesis’s most speculative commitment, and the one on which least direct evidence bears.

#### Difficulty of empirical testing

The six predictions vary in how directly they can be tested with current methods, as the Testability discussion above sets out. Prediction 3 rests on a thin empirical base, anchored by a small number of documented cases rather than convergent evidence; a systematic search for additional coupling mechanisms of the kind Kishimoto et al. (2017) identified has not, to our knowledge, been undertaken, and such mechanisms may prove rare rather than general. Several comparative claims (the cross-clade generality of Prediction 2, the architectural-centrality claim in Prediction 1) require systematic comparative analysis across taxa not yet carried out at the needed scale.

#### Alternative explanations

The architectural account has been assessed against specific named alternatives, gradient-selection accounts, standard genetic-inheritance accounts, component-level accounts of disease, and the Extended Evolutionary Synthesis, rather than a single undifferentiated rival. This is a strength but also a limitation: each prediction’s discriminating force depends on the alternative being correctly characterised, and a more sophisticated version of any of these might absorb a given pattern without requiring the architectural level proposed here. The selection-for-robustness account under Prediction 1 is the clearest instance already engaged directly in the text; similarly sophisticated versions of the others may exist and have not all been anticipated.

#### Operationalising architecture

Treating CHORA as an organisational level resolves a conceptual question but not a practical one: architectural centrality, coupling, and entrenchment lack agreed quantitative measures allowing the precision available to, say, sequence-based measures of conservation. Where this paper speaks of components as architecturally central or peripheral, or architectures as coupled or independently instantiated, these distinctions are drawn from the qualitative pattern of evidence reviewed in Part I and Section 1, not an independently validated metric. Developing such a metric, so centrality and coupling could be assessed directly rather than inferred from the failure pattern they explain, is among the more pressing tasks for further work.

## Concluding remarks

This paper has developed two complementary tasks. Section 1 set out six predictions following from the four commitments of the CHORA Hypothesis, examined what alternative accounts predict in each case, and identified the kinds of findings that would extend, fail to discriminate, or weigh against each prediction. Section 2 drew out the implications for systemic health: health and entrenched systemic disease as expressions of the same regulatory architecture rather than opposing states defined solely by component function.

The framework developed in Part I and refined through this paper does not replace mechanistic explanations or adaptive analyses. It situates them within a broader account that makes visible what those approaches alone cannot fully capture: the structural logic of breakdown, the conditions of entrenchment, the continuity across developmental and intergenerational scales, and the possibility that apparently distinct pathologies share deeper organisational features. The empirical case for the hypothesis rests on the unifying force of the predictions developed here, the limitations acknowledged above, and whether the account proves capable of organising further observations as the relevant literature develop.

The hypothesis advanced here has consequences beyond the empirical and health-relevant domains addressed in this paper and in Part I: for the relation between selection and the architectural conditions of its operation, the kind of explanation appropriate to the persistence of organised biological function across deep time, and the genealogy of organisational thinking within which the framework sits. These conceptual implications are addressed in a separate forthcoming publication.

Whether it proves adequate is an empirical and conceptual question. But the proposal is definite. One and the same regulatory architecture sustains coherence across the lineage and the organism, and produces the convergent forms of systemic disease when its operation within the organism fails. The predictions developed here are what make it answerable to evidence.

## Data Availability

The original contributions presented in the study are included in the article/supplementary material, further inquiries can be directed to the corresponding author.
